# Achievement Emotions Questionnaire–Elementary School: Psychometric Analysis of a Modified Version (AEQ-ES-Gn)

**DOI:** 10.3390/bs16101928

**Published:** 2026-10-17

**Authors:** Francisco Peixoto, Jelena Radišić, Kajsa Yang Hansen, Xin Liu, Anu Laine, Aleksandar Baucal, Äli Leijen, Joana Pipa

**Affiliations:** 1 EDUNOVA.ISPA|Interdisciplinary Research Centre in Education, ISPA—Instituto Universitário, 1149-041 Lisbon, Portugal; jpipa@ispa.pt; 2 Department of Teacher Education and School Research, Faculty of Educational Sciences, University of Oslo, 0317 Oslo, Norway; jelena.radisic@ils.uio.no; 3 Department of Education and Special Education, University of Gothenburg, SE-405 30 Gothenburg, Sweden; kajsa.yang-hansen@ped.gu.se; 4 College of Teacher Education, Ningbo University, Ningbo 315211, China; liuxin2@nbu.edu.cn; 5 Faculty of Educational Sciences, University of Helsinki, 00014 Helsinki, Finland; anu.laine@helsinki.fi; 6 Faculty of Philosophy, University of Belgrade, 11000 Belgrade, Serbia; abaucal@f.bg.ac.rs; 7 Institute of Education, University of Tartu, 50411 Tartu, Estonia; ali.leijen@ut.ee

**Keywords:** achievement emotions, AEQ-ES, elementary school students, factorial validity, measurement invariance, temporal stability

## Abstract

The Achievement Emotions Questionnaire–Elementary School uses pictorial response anchors to assess enjoyment, boredom, and anxiety across academic settings. This study examined the psychometric properties of a modified mathematics version, the AEQ-ES-Gn, in which the original gender-specific faces were replaced by gender-neutral, smiley-based representations. Participants were 12,738 students (*M* age at Wave 1 = 9.57 years, *SD* = 0.83; 50.7% girls among students with available gender information) from 287 schools in Estonia, Finland, Norway, Portugal, Serbia and Sweden. Students were in grades 3–4 at Wave 1 and Grades 4–5 approximately one year later in Wave 2. Confirmatory factor analyses compared alternative measurement models, followed by tests of longitudinal and multigroup invariance, reliability, convergent and discriminant validity, temporal stability, and associations with mathematics competence. A hierarchical model distinguishing emotions and academic settings was retained, showing a good fit and providing the most appropriate overall solution for subsequent analyses. Scalar invariance was supported over time and across gender, grade, and country. Scores showed adequate-to-excellent reliability, generally satisfactory convergent validity, and evidence of discriminant validity, although enjoyment and boredom were strongly negatively related. Temporal stability was moderate, and associations with mathematics competence were small and theoretically consistent. These findings support the AEQ-ES-Gn as a reliable and valid measure of mathematics-related achievement emotions among students in Grades 3–5, while direct comparisons with the original response format remain necessary.

## 1. Introduction

Emotions are an integral part of students’ everyday experiences at school. Children may enjoy engaging with a challenging learning task, experience anxiety when anticipating an assessment, or feel bored during an unengaging lesson. These emotional experiences are closely intertwined with students’ motivation, self-regulation, engagement, and achievement (Camacho-Morles et al., 2021; Forsblom et al., 2022; Pekrun, 2006, 2024; Pekrun et al., 2002). Accordingly, the past two decades have witnessed a substantial increase in research on emotions in educational contexts, shifting the field beyond its traditional focus on test anxiety towards a broader consideration of the diversity of emotions experienced during learning and achievement activities (Camacho-Morles et al., 2021; Pekrun, 2024).

Achievement emotions are increasingly recognised as central to students’ motivation, learning, and academic achievement. The growing scholarly interest in this area is evident in the expanding body of research on achievement emotions. A search of the Web of Science database using the term achievement emotions indicated a marked increase in publications, from 175 papers in the 1990s to 5637 papers in the last five years. Understanding these emotions is particularly important in elementary education, as children’s early emotional experiences in school can shape the development of motivational beliefs, learning behaviours, and attitudes toward academic domains (Lichtenfeld et al., 2012, 2023; Pekrun, 2006; Raccanello et al., 2019).

Consequently, educational research has devoted growing attention to understanding students’ emotional experiences and to developing theoretically grounded instruments capable of assessing these emotions across different educational stages. In this regard, several well-established measures are available for adolescents and adults (Achievement Emotions Questionnaire [AEQ]; Pekrun, 2024; Pekrun et al., 2011). In contrast, the Achievement Emotions Questionnaire–Elementary School (AEQ-ES; Lichtenfeld et al., 2012) was developed to provide a developmentally appropriate measure of children’s achievement emotions. A distinctive feature of the AEQ-ES is the use of pictorial response anchors to facilitate students’ responses. However, because these anchors portray either boys or girls, they may not promote identification among all students.

Against this background, the present study examines an adapted version of the AEQ-ES in which the original gender-specific pictorial response anchors were replaced by neutral smiley-based representations, referred to as the AEQ-ES-Gn. Although the emotional constructs, item wording, and response scale remain unchanged, the pictorial response anchors constitute an integral component of questionnaire administration in childhood, guiding how children interpret and use the response categories (Raccanello & Burro, 2024). Thus, it is important to establish whether this modification preserves the psychometric quality of the instrument.

Accordingly, the present study evaluates the factorial validity, reliability, and measurement invariance of the AEQ-ES-Gn across gender, grade level, six European countries, and over time. By examining whether the modified response format functions equivalently across these groups and occasions, this study contributes to the availability of developmentally appropriate and inclusive instruments for assessing achievement emotions in elementary school children and provides further evidence supporting meaningful cross-group and longitudinal comparisons.

### 1.1. Achievement Emotions

Achievement settings, and classrooms in particular, are inherently emotional contexts (Pekrun, 2024). Learning new content, solving difficult problems, participating in classroom activities, completing homework, and taking tests can elicit a wide range of emotional responses. Achievement emotions refer specifically to emotions directly tied to achievement-related activities or their outcomes (Pekrun, 2006; Pekrun et al., 2002). Activity emotions concern ongoing achievement processes, such as enjoyment or boredom while learning, whereas outcome emotions relate to anticipated or past success and failure, such as hope, anxiety or pride (Pekrun, 2024).

According to the three-dimensional taxonomy proposed by Pekrun et al. (2023), achievement emotions can be differentiated according to three dimensions: valence, arousal, and object focus. Valence distinguishes positive and pleasant from negative and unpleasant emotions. For example, a student may enjoy working on a project during class (positive valence) and feel angry about the outcome resulting from another school-related task. Arousal differentiates physiologically activating from deactivating emotions. A student may feel excited about the new school project (high arousal), while feeling bored (low arousal) regarding a specific subject’s topic. Object focus distinguishes emotions directed towards ongoing activities, such as enjoyment and frustration while solving school-related tasks, from those concerning prospective or retrospective achievement outcomes. These include, for example, a student feeling anxious before a test (prospective), or being proud of solving an important task (retrospective). This framework demonstrates why individual emotions should not simply be subsumed under general categories of positive and negative affect (Pekrun et al., 2023).

### 1.2. The Control-Value Theory of Achievement Emotions

The control-value theory of achievement emotions (CVT; Pekrun, 2006, 2024; Pekrun & Perry, 2014) provides the main theoretical framework for understanding why emotions arise and how they affect learning. Its central proposition is that achievement emotions are primarily shaped by the interplay between two types of cognitive appraisals: perceived control and perceived value. Control concerns students’ perceived competence to influence an achievement activity or outcome, whereas value concerns the perceived importance or intrinsic value of that activity or outcome (Pekrun, 2024).

Different combinations of control and value appraisals are associated with different emotions (Forsblom et al., 2022; Pekrun, 2006). Enjoyment is likely when students value a task and perceive themselves as competent to master that task. Boredom, in contrast, may emerge when an activity lacks subjective value or when its demands are poorly matched with students’ perceived competences. Anxiety typically arises when an important negative outcome or failure is perceived as possible and control over the outcome is uncertain or low (Pekrun, 2006).

The CVT also proposes that emotions affect students’ learning and achievement (Barroso et al., 2021; Camacho-Morles et al., 2021; Forsblom et al., 2022; Lichtenfeld et al., 2023; Symes et al., 2025). In this regard, whereas positive activating emotions have generally been found to be positively associated with achievement in different subjects, negative emotions are negatively associated with students’ achievement (Lichtenfeld et al., 2023; Symes et al., 2025).

Importantly, the relationships between appraisals, emotions, and achievement are reciprocal (Forsblom et al., 2022; Pekrun, 2024). Success may strengthen perceived control and enjoyment, which promote further engagement and achievement. Conversely, repeated difficulties may reduce perceived control, increase anxiety or boredom, and interfere with subsequent learning. Longitudinal research with elementary school children supports these reciprocal dynamics, showing that enjoyment positively predicts subsequent mathematics achievement and is itself predicted by previous achievement, whereas anxiety and boredom are reciprocally associated with poorer performance (Lichtenfeld et al., 2023).

The present study deliberately adopts the achievement-emotion perspective of CVT rather than seeking to provide a comprehensive account of children’s emotional experiences at school. This theoretical focus aligns with the study’s purpose: to evaluate the psychometric functioning of a modified version of an instrument developed within the CVT tradition. Other conceptualisations of emotion may capture additional aspects of children’s school experiences, but evaluating those frameworks lies beyond the scope of the present validation study.

### 1.3. The Assessment of Achievement Emotions

The development of theoretically grounded instruments has been fundamental to advances in achievement-emotion research. Pekrun et al. (2011) developed the Achievement Emotions Questionnaire (AEQ), which assesses nine discrete achievement emotions—enjoyment, hope, pride, relief, anger, anxiety, shame, hopelessness, and boredom—across three academic settings: attending class, learning, and taking tests. By assessing emotions within specific achievement contexts, the AEQ reflects the central assumption of the CVT that achievement emotions are context-specific (Pekrun et al., 2011). More recently, the Achievement Emotions Questionnaire–Revised (AEQ-R) was introduced to align the instrument with recent theoretical developments, particularly the expanded conceptualisation and taxonomy of achievement emotions (Pekrun et al., 2023). Despite these advances, both instruments were developed for older students, highlighting the need for developmentally appropriate measures for younger school students. Elementary school students differ from older students in their reading ability, emotional vocabulary, metacognitive development, and capacity to distinguish between complex affective experiences. Consequently, assessing achievement emotions in younger populations requires developmentally appropriate wording, shorter and clearer items, and a structure that reflects children’s everyday academic experiences. Assessing achievement emotions in younger children also poses specific methodological challenges. Compared with older students, younger children have a more limited capacity to integrate experiences across situations and occasions into generalised self-reports. Broad or trait-like questions may therefore place greater demands on memory, abstraction, and the ability to summarise recurring emotional experiences. Developmentally adapted measures have addressed these constraints by using brief, concrete item wording, familiar and clearly specified contexts, relatively simple response formats, and, in some cases, pictorial support. At the same time, pictorial representations are not developmentally neutral: children’s ability to recognise and interpret emotional expressions develops with age, making it important to examine visual supports in relation to the intended age group (Raccanello & Burro, 2024).

Against this background, recent instruments illustrate complementary approaches to assessing affective experiences among younger students. The Achievement Emotions Questionnaire–Writing in Early Elementary School (AEQ-WEES; Schlesier et al., 2026), for example, assesses achievement emotions in the specific context of early writing acquisition. Related measures address broader or conceptually distinct affective experiences, including school-related well-being and happiness (HIFAMS; Allen et al., 2018) and school fears related to academic failure and evaluation (SFSS-II; García-Fernández et al., 2010). Within this broader measurement landscape, the AEQ-ES focuses specifically on theoretically defined achievement emotions across different achievement settings.

### 1.4. The Achievement Emotions Questionnaire for Elementary Students (AEQ-ES)

To address the aforementioned limitations, Lichtenfeld et al. (2012) developed the Achievement Emotions Questionnaire–Elementary School (AEQ-ES) specifically for elementary school students. The selection of these three emotions reflects the established scope of the AEQ-ES rather than an attempt to represent the full range of achievement emotions. The AEQ-ES assesses achievement emotions of enjoyment, anxiety, and boredom across three achievement settings: attending class, completing homework, and taking tests. The focus on these emotions is justified by their prominence in achievement settings (Barroso et al., 2021; Camacho-Morles et al., 2021; Lichtenfeld et al., 2012, 2023) and by their representation of distinct positions within the three-dimensional taxonomy of achievement emotions (Pekrun et al., 2023). Enjoyment is a positive activating activity emotion; boredom is a negative deactivating activity emotion; and anxiety is a negative activating emotion that is typically associated with anticipated failure. Their different positions within the taxonomy imply different cognitive antecedents and educational consequences. Examining these three emotions therefore provides meaningful coverage of positive and negative as well as activating and deactivating emotional experiences that are particularly salient in elementary school settings (Lichtenfeld et al., 2012; Pekrun et al., 2023). Accordingly, the inclusion of only one positive emotion reflects the design and scope of the original instrument. In the present study, this three-emotion structure was retained because the modification concerns the pictorial response format rather than the AEQ-ES’s conceptual content; adding another positive emotion, such as pride or hope, would constitute an extension of the instrument.

The AEQ-ES was adapted to assess achievement emotions in the specific context of mathematics, with the assumption that control and value appraisals and, consequently, achievement emotions are domain-specific (Bieleke et al., 2023; Goetz et al., 2007). In their study, Lichtenfeld et al.’s (2012) findings provided support for the reliability and validity of the AEQ-ES. Specifically, the best-fitting model was a hierarchical structure comprising three second-order factors (enjoyment, anxiety, and boredom), each reflected by setting-specific first-order factors corresponding to class, learning, and test contexts. The instrument also demonstrated the expected associations with students’ control and value appraisals, providing evidence of its construct validity (Lichtenfeld et al., 2012).

Furthermore, no significant gender differences were observed, supporting equivalence of emotional patterns across boys and girls. The study also examined the cross-cultural applicability of the AEQ-ES by comparing German and U.S. elementary school students. The findings supported measurement invariance across the two cultural contexts, indicating that the instrument assessed the three achievement emotions equivalently in both countries (Lichtenfeld et al., 2012).

Subsequent studies have extended this evidence across languages and cultural contexts. Raccanello et al. (2019) found that the hierarchical structure of the AEQ-ES was invariant across Italy, Germany, and the United States, as well as across gender, grades, and mathematics versus native-language domains. A Japanese adaptation also demonstrated satisfactory psychometric properties and confirmed the hierarchical structure proposed by Lichtenfeld et al. (2012) and demonstrated theoretically consistent relations with control and value appraisals and students’ intrinsic motivation (Suzuki & Tonegawa, 2022). In Portugal, the study by Sanches et al. (2020) supported the hierarchical structure of the AEQ-ES and measurement invariance across gender and grades. The measure also demonstrated good reliability and temporal stability, while correlations with perceived competence and intrinsic motivation provided evidence of external validity.

One feature of questionnaires designed for younger children concerns the use of pictorial response supports. Such supports can reduce demands associated with verbal or numerical response formats, enhance access to semantic meaning, and help children map their responses onto different levels of a rating scale (Raccanello & Burro, 2024). However, the design of pictorial anchors also requires consideration of whether the representations are equally accessible and appropriate for all respondents. Evidence from recent work on pictorial assessment of achievement emotions illustrates both the potential value of visual support and the importance of considering children’s age and ability to interpret emotional representations (Raccanello & Burro, 2024).

The AEQ-ES is generally accompanied by a pictorial response anchor, representing either boys’ or girls’ faces, designed to facilitate children’s use of the response scale. However, the characteristics of the figures used in the pictorial support may affect how respondents relate to and interpret the response options. Although such gender-specific representations may facilitate identification for some children, they may not provide equally inclusive support for students who do not identify with either representation. More generally, replacing gender-specific figures with non-gendered pictorial anchors allows the same response format to be administered to all students, without requiring children to identify with a male or female figure or to select a gender-specific version of the instrument. A common response format may therefore facilitate administration while avoiding the explicit linking of the response format to respondent gender. However, whether this modification preserves equivalent measurement functioning across boys and girls requires empirical examination.

### 1.5. The Present Study

The present study aimed to assess the psychometric properties of the AEQ-ES-Gn, a modified version of the Achievement Emotions Questionnaire–Elementary School (AEQ-ES; Lichtenfeld et al., 2012). The modification concerns the pictorial response anchors, with the original representations of boys’ and girls’ faces replaced by more general smiley-based representations.

The present validation concerns students in Grades 3–5, broadly corresponding to the middle-to-later years of primary education in the participating systems. Because the organisation and duration of primary schooling vary across countries, grade level is used here to define the developmental scope of the evidence, rather than assuming that the labels primary or elementary school denote an equivalent age range across systems.

Previous research has supported the structural validity, reliability, and external validity of the AEQ-ES, demonstrating its usefulness for assessing young children’s achievement emotions across different academic settings (Raccanello et al., 2019; Sanches et al., 2020; Suzuki & Tonegawa, 2022). Across these adaptations, the scale has retained pictorial response anchors depicting boys’ and girls’ faces to facilitate children’s use of the response scale. However, this pictorial support may not promote identification among all students, particularly those who do not identify with these binary gender representations.

In the present study, neutral smiley-based representations were used to provide a response format that does not require children to identify with either a male or female figure and may therefore be more inclusive for students who do not identify with either pictorial representation.

Importantly, although no changes were made to the emotion dimensions or item wording, the modified pictorial response format may affect how respondents interpret and use the response scale (Raccanello & Burro, 2024), justifying the need to empirically re-examine the psychometric properties of the AEQ-ES-Gn.

Additionally, this study aimed to investigate the measurement invariance of the AEQ-ES-Gn across gender, grade levels, cultural contexts, and time. Gender invariance is particularly relevant. Previous studies have supported gender invariance for the AEQ-ES (Raccanello et al., 2019; Sanches et al., 2020). The replacement of gender-specific pictorial anchors with a common smiley-based anchor provides an additional rationale for examining whether the instrument operates equivalently across genders.

Grade invariance is important because elementary school encompasses considerable development in vocabulary, reading skills, and emotional understanding. Previous research has supported AEQ-ES invariance across second and fourth grades and across Portuguese third- and fourth-grade students (Raccanello et al., 2019; Sanches et al., 2020). Nevertheless, equivalence should be re-examined after modifying the response format, particularly when the scale is administered to students of different ages.

Cross-country invariance is required when comparing children from different cultural or linguistic contexts. Previous research has provided encouraging evidence for the AEQ-ES, including equivalence across German and American samples and across Italy, Germany, and the United States (Lichtenfeld et al., 2012; Raccanello et al., 2019). Establishing invariance in additional cultural contexts strengthens evidence that enjoyment, anxiety, and boredom are measured comparably across populations. The present study adds to this evidence by assessing measurement invariance across six different European countries.

Finally, longitudinal invariance is necessary for interpreting emotional change over time (Forsblom et al., 2022; Lichtenfeld et al., 2023). This is especially important given evidence that achievement emotions and performance reciprocally influence one another across the elementary school years (Lichtenfeld et al., 2023).

Taken together, the present study evaluates whether the established psychometric structure of the AEQ-ES is retained when the original gender-specific pictorial response anchors are replaced by a common smiley-based format. Mathematics competence was included as an external criterion rather than as part of the AEQ-ES-Gn measurement model. Based on control-value theory and previous AEQ-ES findings, enjoyment was expected to be positively associated with mathematics competence, whereas boredom and anxiety were expected to show negative associations. These associations were used to examine criterion-related validity rather than to test causal effects of achievement emotions on performance.

Accordingly, the study addressed four research questions. RQ1 concerned the cross-sectional psychometric functioning of the AEQ-ES-Gn at each wave: whether the established hierarchical structure was retained and whether the resulting dimensions demonstrated adequate reliability, convergent validity, and discriminant validity. Based on previous AEQ-ES validation studies, we expected the hierarchical structure distinguishing emotion and academic setting to provide the most appropriate representation of the data.

RQ2 concerned cross-sectional measurement invariance across gender, grade, and country. Based on previous evidence for the AEQ-ES, we expected measurement invariance across these groups.

RQ3 concerned longitudinal functioning: whether the AEQ-ES-Gn demonstrated measurement invariance across the two occasions and whether corresponding emotion dimensions showed temporal stability over approximately one year.

RQ4 concerned criterion-related validity. We expected enjoyment to be positively associated with mathematics competence and boredom and anxiety to be negatively associated with mathematics competence, consistent with control-value theory and previous empirical findings.

## 2. Materials and Methods

### 2.1. Participants

Participants were 12,738 students from 761 classes across 287 schools in six European countries. Schools were approached as the recruitment unit and asked to contribute one intact classroom per participating grade. All participants completed at least one item of the Achievement Emotions Questionnaire–Elementary School (AEQ-ES) at one or both measurement occasions. Participants were from Estonia (*n* = 1924; 15.1%), Finland (*n* = 2014; 15.8%), Norway (*n* = 2290; 18.0%), Portugal (*n* = 2133; 16.7%), Serbia (*n* = 2160; 17.0%), and Sweden (*n* = 2217; 17.4%). Gender information was available for 12,704 students: 6436 (50.7%) were girls and 6268 (49.3%) were boys; gender information was missing for 34 students.

At Wave 1, students attended either Grade 3 (*n* = 6195; 48.6%) or Grade 4 (*n* = 6543; 51.4%). Their mean age was 9.57 years (SD = 0.83, range = 7–14 years; age data available for n = 11,579). At Wave 2, approximately one year later, the students were in Grades 4 (*n* = 6215; 48.8%) and 5 (*n* = 6523; 51.2%), respectively. Their mean age was 10.55 years (SD = 0.83, range = 8–14 years; age data available for *n* = 10,465). The present validation therefore concerns students in Grades 3–5. No students from Grades 1–2 were included, and the present findings should not be interpreted as evidence regarding the functioning of the modified response format in those grades.

AEQ-ES-Gn data were available for 11,690 students at Wave 1 and 10,547 students at Wave 2. In total, 9499 students provided AEQ-ES-Gn data at both waves, whereas 2191 contributed data only at Wave 1 and 1048 only at Wave 2. The analyses therefore used all available information from the 12,738 students who contributed AEQ-ES-Gn data on at least one measurement occasion, rather than being restricted to students with complete data at both waves.

Ethical approval was obtained from the appropriate institutional or national bodies in each participating country, and all study procedures complied with the principles of the Declaration of Helsinki and its subsequent revisions. Students participated voluntarily and were included only after written informed consent had been provided by their parents or legal guardians.

### 2.2. Measures

#### 2.2.1. Achievement Emotions Questionnaire—Elementary School

Students’ mathematics-related achievement emotions were assessed using a modified version of the Achievement Emotions Questionnaire–Elementary School (AEQ-ES; Lichtenfeld et al., 2012), referred to as the AEQ-ES-Gn. Whereas the original AEQ-ES uses separate pictorial response formats depicting boys or girls, the AEQ-ES-Gn uses a common non-gender-specific format for all students. In the original versions, enjoyment, boredom, and anxiety are each represented by a distinct sequence of five facial expressions that increase in intensity across the five response categories. The AEQ-ES-Gn retained this emotion-specific gradation: separate sequences of neutral faces were developed for enjoyment, boredom, and anxiety, following the same progression from low to high emotional intensity as in the original pictorial format. The neutral facial sequences were developed by the project team, revised following discussion within the project group, and subsequently reviewed by the project’s external advisory group with regard to the representation of the intended emotions and their progression in intensity. The same five-face sequence was used for all items assessing the corresponding emotion.

**Figure 1:**
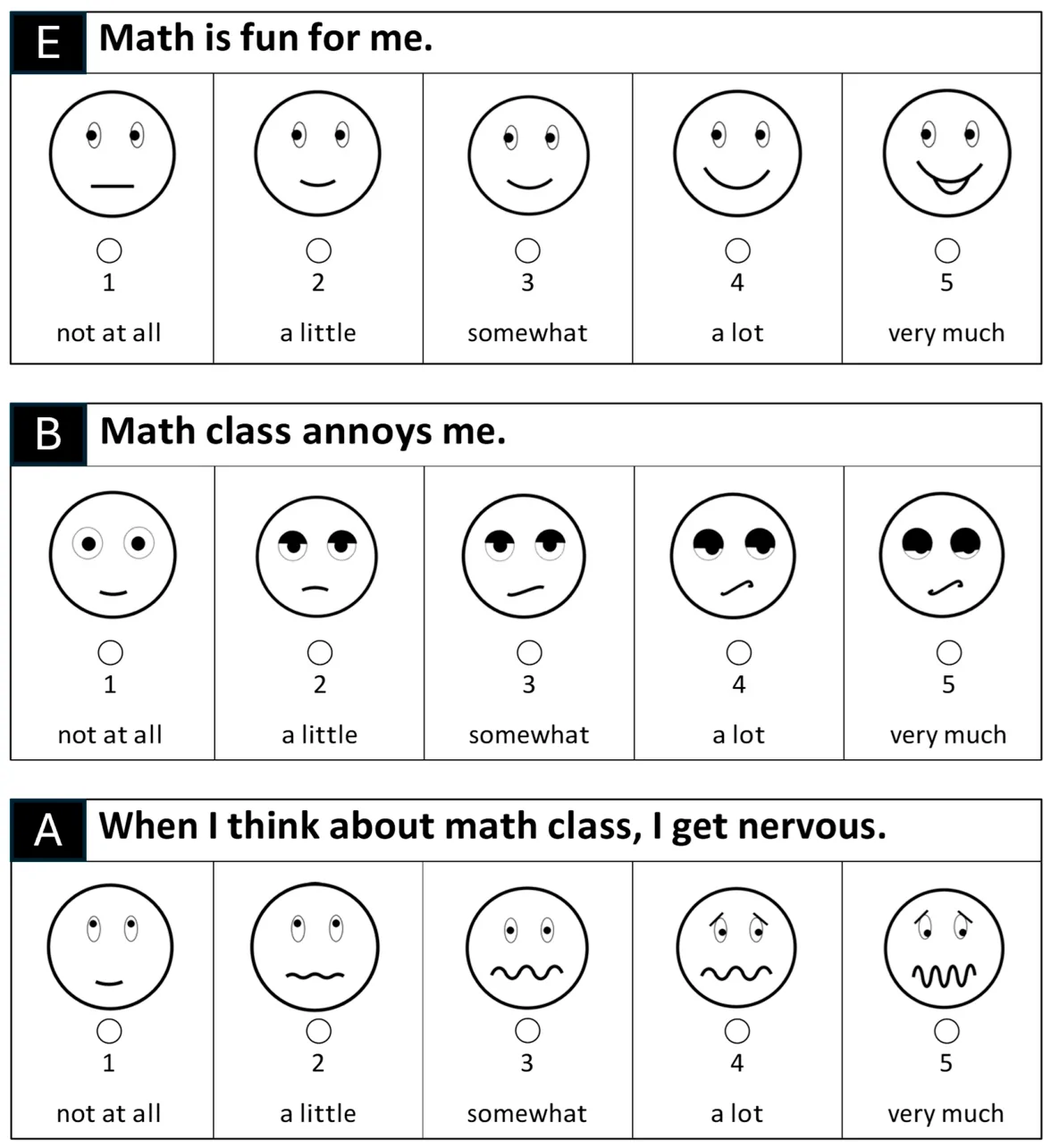
Emotion-specific pictorial response anchors used in the AEQ-ES-Gn for enjoyment (E), boredom (B), and anxiety (A). Within each emotion, the five facial expressions correspond to increasing response intensity from 1 (not at all) to 5 (very much).

Consistent with the original AEQ-ES, the modified version retained the three emotion dimensions of enjoyment, boredom, and anxiety and the original item content; the present modification concerned the pictorial response format rather than the conceptual scope of the instrument (Figure 1).

The instrument comprises 28 items assessing enjoyment (9 items), boredom (7 items), and anxiety (12 items) across three achievement contexts: mathematics classes (12 items), learning activities (8 items), and tests (8 items). Boredom is assessed in relation to classes and learning activities, whereas enjoyment and anxiety are assessed across all three contexts. Students indicate how well each statement describes their experience using a five-point pictorial response scale, with corresponding emotion-specific facial sequence representing response intensity from 1 (not at all) to 5 (very much). Example items include “I enjoy math class” (enjoyment), “I find doing math boring” (boredom), and “When I take a math test, I am afraid of doing poorly” (anxiety). Responses are averaged within each subscale, with higher scores indicating a greater intensity of the respective emotion.

All AEQ-ES items were translated from English into the predominant language of each participating country and then back-translated into English. The back-translated versions revealed no substantive differences in content from the original English wording.

#### 2.2.2. Mathematics Competence

Mathematics competence was assessed at both waves using two complementary measures of mathematical problem solving and arithmetic fluency. The first measure was a curriculum-based mathematics test constructed from released Grade 4 items from the 2011 cycle of the Trends in International Mathematics and Science Study (TIMSS; Mullis et al., 2012), used with permission from the International Association for the Evaluation of Educational Achievement (IEA; Approval IEA-22-022). The items covered the TIMSS content domains of number, geometric shapes and measures, and data display. The test additionally assessed the cognitive domains of knowing, applying, and reasoning. At Wave 1, the Grade 3 form comprised 12 items and the Grade 4 form comprised 14 items, with seven common linking items. At Wave 2, the Grade 4 and Grade 5 forms each comprised 14 items and shared nine linking items. The Grade 4 form was identical across the two waves, thereby providing an additional basis for longitudinal linking. Students had 30 min to complete the test. Each correct response received one point; therefore, maximum raw scores were 12 for the Grade 3 form and 14 for the Grade 4 and Grade 5 forms. Rasch-scaled scores were used in the analyses to account for differences in item composition and difficulty across forms and to support comparisons across grades and waves.

Arithmetic fluency was assessed using the calculation test developed by Klausen and Reikerås (2016). Each subtest comprised 45 single-operation calculations arranged in three columns, and students were instructed to work through the items sequentially and solve as many as possible within two minutes. At Wave 1, Grade 3 students completed the addition and subtraction subtests, whereas Grade 4 students also completed the multiplication subtest. At Wave 2, students in both Grades 4 and 5 completed all three subtests—addition, subtraction, and multiplication. One point was awarded for each correct response, resulting in a maximum score of 45 per operation. Thus, the maximum total score was 90 for Grade 3 students at Wave 1 and 135 for students completing all three subtests. Scores for addition, subtraction, and multiplication were retained separately, allowing performance on the operations administered at each grade level and measurement occasion to be distinguished.

### 2.3. Data Analyses

To examine the factorial structure of the scale, confirmatory factor analyses (CFAs) were conducted in Mplus 9.1. A confirmatory rather than exploratory approach was adopted because the AEQ-ES has an established theoretically specified measurement structure that has been supported in previous validation studies. The present study aimed to examine whether this established structure was retained following modification of the pictorial response format, rather than to derive a new dimensional structure.

**Figure 2:**
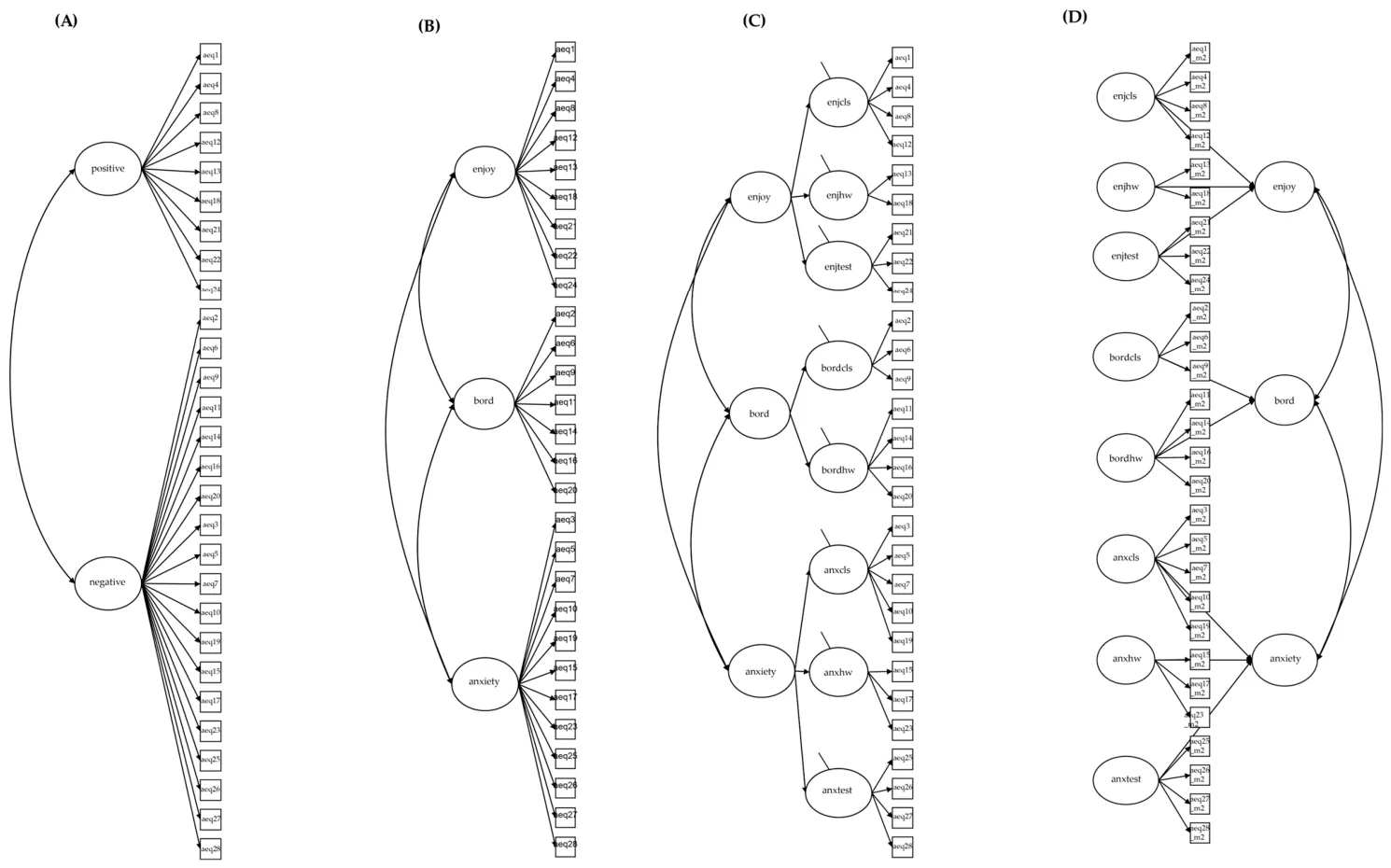
Alternative measurement models tested to examine the factorial structure of the AEQ-ES-Gn. (**A**) Two-factor model; (**B**) Three-factor model; (**C**) Hierarchical model; (**D**) Bi-factor model.

Four alternative models informed by previous findings (e.g., Lichtenfeld et al., 2012; Raccanello et al., 2019; Sanches et al., 2020; Suzuki & Tonegawa, 2022) were tested to examine the factorial structure of the AEQ-ES-GN separately at Wave 1 and Wave 2 (Figure 2). The first model (Figure 2A) was a correlated two-factor model in which the 28 items loaded on one of two factors, depending on whether they assessed a positive or negative emotion. The second model (Figure 2B) was a correlated three-factor model in which the 28 items loaded on one of three emotion factors: enjoyment, boredom, or anxiety. In this model, items referring to classes, learning, and tests were treated as indicators of the same emotion factor, and the three emotion factors were allowed to correlate.

The third model (Figure 2C) was a hierarchical model that distinguished between the emotional domain and the context in which the emotion was experienced. At the first-order level, separate factors were specified for class-related, learning-related, and test-related emotions. Specifically, enjoyment and anxiety were each represented by three first-order factors corresponding to classes, learning, and tests. Boredom was represented by two first-order factors—class-related and learning-related boredom—because the instrument did not include items assessing test-related boredom. At the second-order level, the context-specific factors loaded on their respective higher-order enjoyment, boredom, or anxiety factors. The three higher-order emotion factors were allowed to correlate.

The fourth model (Figure 2D) was a bifactor-like model in which each item loaded simultaneously on a general emotion factor and a context-specific factor. Thus, each item loaded on the corresponding general enjoyment, boredom, or anxiety factor and, concurrently, on a specific class, learning, or test factor. The context-specific factors captured common variance associated with the situational context after accounting for the corresponding general emotion factor. All context-specific factors were specified as mutually orthogonal and as orthogonal to the three general emotion factors, whereas the general emotion factors were allowed to correlate. In addition, the loadings of the two items defining the specific learning-related enjoyment factor were constrained to equality. The three loadings defining the specific learning-related anxiety factor were also constrained to equality because preliminary estimation indicated local identification problems involving this factor.

With five response categories and the large sample size, items were treated as approximately continuous and models were estimated using the robust maximum likelihood (MLR) estimator. The TYPE = COMPLEX option in Mplus was used to account for the non-independence of observations within schools. School was specified as the clustering variable in line with the sampling design, in which schools were treated as the recruitment unit and asked to contribute one intact classroom per participating grade. School-level clustering therefore accounts for dependence among students within the same school, including those sharing a classroom. Given the well-documented sensitivity of the chi-square statistic to sample size (Kline, 2016), model fit was evaluated through a combination of fit indices: the Comparative Fit Index (CFI), the Tucker–Lewis Index (TLI), and the Root Mean Square Error of Approximation (RMSEA). CFI and TLI values exceeding 0.90 were interpreted as reflecting adequate fit, whereas values above 0.95 were taken to denote very good fit (Schumacker & Lomax, 2016). Regarding RMSEA, values below 0.08 were treated as evidence of good fit, and values under 0.05 as indicative of very good fit (Hu & Bentler, 1999; Schumacker & Lomax, 2016). Under MLR estimation, incomplete observations were retained using full-information maximum likelihood, thereby allowing all available data to contribute to model estimation.

In addition to examining the scale’s structure, we evaluated its reliability, convergent validity and discriminant validity. Reliability coefficients above 0.70 were deemed acceptable (DeVellis & Thorpe, 2022; Nunnally, 1978; Nunnally & Bernstein, 1994). The assessment of convergent and discriminant validity followed the criteria originally established by Fornell and Larcker (1981) and further refined by Cheung and Chang (2017). Specifically, convergent validity was supported when the average variance extracted (AVE) surpassed 0.50, provided that composite reliability also exceeded 0.70 and that factor loadings were not substantially below 0.50 (Cheung & Chang, 2017). Discriminant validity was supported when the squared correlation between any two dimensions of the scale was lower than the AVE of each corresponding factor. Cheung and Chang (2017) further recommended that inter-construct correlations should remain below 0.70. Where the enjoyment–boredom correlation exceeded this recommended threshold, an additional theoretically targeted model was estimated in which enjoyment and boredom were combined into a single higher-order factor and its fit was compared with that of the three-emotion hierarchical model.

Measurement invariance was tested longitudinally and across gender, country, and grade by sequentially comparing configural, metric, and scalar models. Metric invariance was evaluated by constraining the first- and second-order factor loadings to equality, whereas scalar invariance additionally constrained the item intercepts. Following Chen (2007), invariance was considered supported when the decrease in CFI did not exceed 0.010 and the increase in RMSEA did not exceed 0.015. Finally, we examined temporal stability by inspecting the correlations between the AEQ-ES-Gn dimensions across the two waves, and criterion-related validity by relating these dimensions to measures of academic competence.

## 3. Results

**Figure 3:**
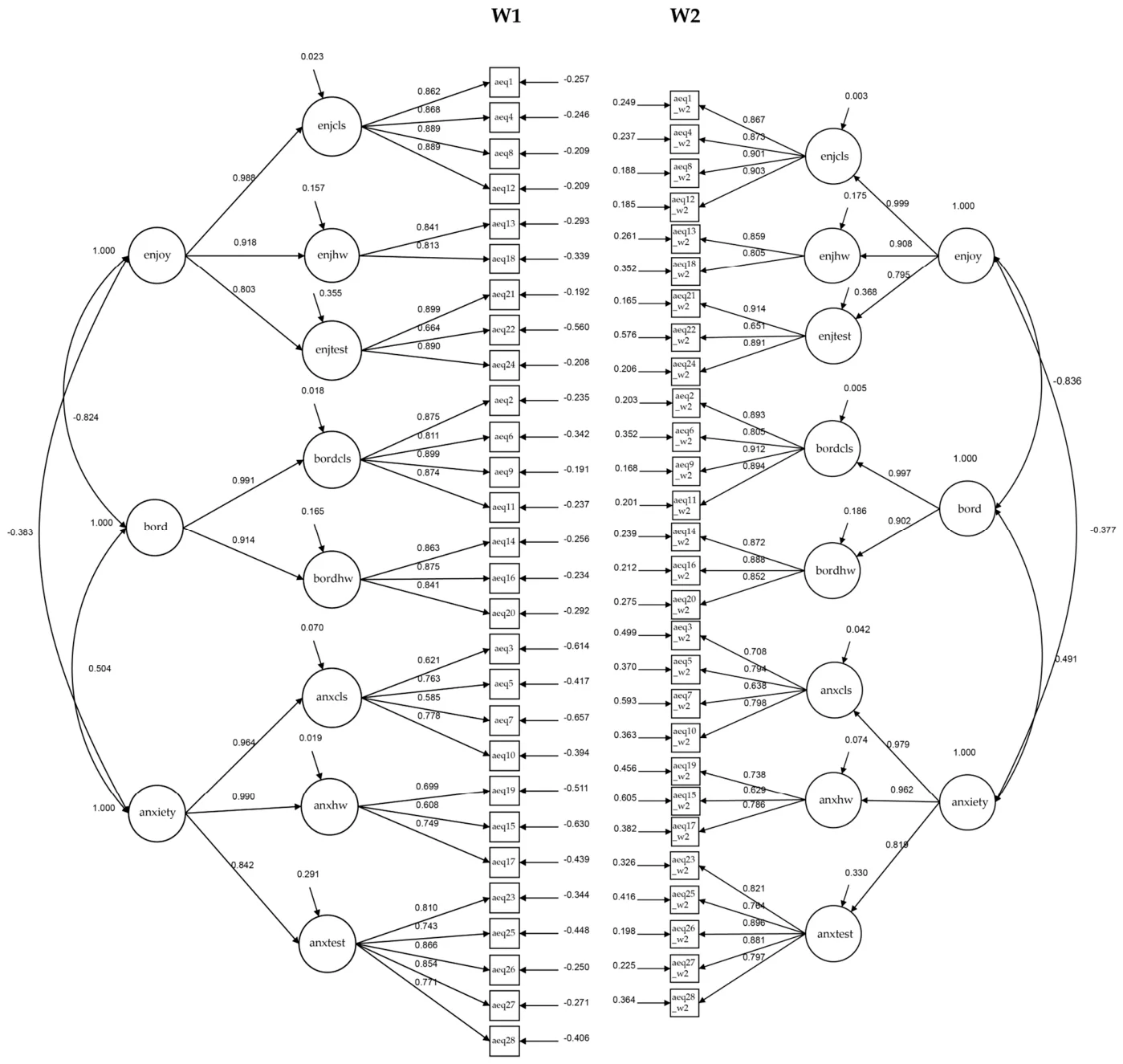
Standardized CFA Results for the Hierarchical AEQ-ES-GN Model at Wave 1 (W1) and Wave 2 (W2). Enjoy = enjoyment; Bord = boredom; Anx = anxiety; Cls = class-related; hw = learning-related.

The fit indices for the four alternative measurement models are presented in Table 1. The results indicated that the two-factor and three-factor models provided a poor fit to the data at both waves. In contrast, the hierarchical and bifactor-like models showed a good fit, with the bifactor-like model exhibiting slightly better fit indices at both waves. However, the bifactor-like model yielded several inadmissible solutions in the measurement invariance analyses, and for this reason it was not retained. Consequently, the hierarchical model was retained as the most appropriate model for the subsequent analyses and was used in all subsequent analyses, including the assessment of reliability, convergent validity, and discriminant validity. Figure 3 presents the standardised results for the hierarchical model at both waves.

As shown in Table 2, longitudinal measurement invariance was supported through the scalar level, with only negligible changes in model fit across the configural, metric, and scalar models (|ΔCFI| ≤ 0.001; |ΔRMSEA| = 0.000). Scalar invariance was also supported across gender, country, and grade at both waves, as the changes in CFI and RMSEA remained below the recommended cutoff values (|ΔCFI| ≤ 0.006; |ΔRMSEA| ≤ 0.002). These findings support the equivalence of the factorial structure, factor loadings, and item intercepts over time and across the examined groups.

The AEQ-ES-GN dimensions generally demonstrated adequate to excellent reliability at both waves (Table 3). For the specific dimensions, composite reliability coefficients ranged from 0.73 to 0.93 at Wave 1 and from 0.76 to 0.94 at Wave 2. Cronbach’s alpha and McDonald’s omega coefficients showed a similar pattern, ranging from 0.72 to 0.93 at Wave 1 and from 0.76 to 0.94 at Wave 2. Reliability was generally highest for enjoyment in class, boredom in class, boredom while learning, and test-related anxiety, whereas the lowest coefficients were obtained for anxiety while learning. The higher-order dimensions also demonstrated adequate to excellent reliability, with coefficients ranging from 0.78 to 0.95 across the three reliability indicators and the two waves.

The findings concerning discriminant validity were mixed. As shown in Table 4, the AVE was greater than the squared correlation for every pair of higher-order dimensions, thereby supporting discriminant validity according to the Fornell–Larcker criterion. However, the correlations between enjoyment and boredom exceeded the absolute-value threshold of 0.70 proposed by Cheung and Chang (2017) at both waves. As specified in the analytical strategy, the alternative hierarchical model combining enjoyment and boredom into a single higher-order factor was therefore examined. This model provided a poorer fit than the model with three distinct higher-order factors at both waves: Wave 1, χ^2^ (341) = 10,550.3, *p* < 0.001, CFI = 0.935, TLI = 0.928, RMSEA = 0.051, 95% CI [0.050, 0.051], SRMR = 0.064, AIC = 767,996.5, BIC = 768,681.6; Wave 2, χ^2^ (341) = 11,551.0, *p* < 0.001, CFI = 0.933, TLI = 0.926, RMSEA = 0.056, 95% CI [0.055, 0.057], SRMR = 0.067, AIC = 703,618.6, BIC = 704,294.1. The fit indices for the model with three higher-order factors are reported in Table 1. In addition, manifest and latent correlations between enjoyment and boredom were examined separately within each wave and country to explore potential cross-country differences in the strength of this association. Results showed consistently strong negative associations across all countries. Pearson correlations were high in every country, generally exceeding 0.65, while latent correlations were even stronger, exceeding 0.80 in all cases (see Table S1 in Supplementary Materials). Although the magnitude of the correlations varied significantly across some countries, the overall pattern was highly consistent, indicating a robust negative association between enjoyment and boredom across cultural contexts and measurement waves.

The one-year test–retest correlations were moderate, indicating reasonable temporal stability (Table 5). Enjoyment showed the strongest longitudinal associations at both the specific and higher-order levels. For each specific dimension, the correlation with the corresponding W2 dimension was among its strongest cross-wave associations. In some cases, however, the association with the corresponding higher-order dimension was comparable or slightly stronger. Additionally, regression analyses provided further evidence of temporal stability: when each specific W2 dimension was simultaneously predicted by all eight specific W1 dimensions, the corresponding W1 dimension consistently showed the largest standardised regression coefficient in absolute value (see Tables S2–S9 in Supplementary Materials).

Associations between the emotion dimensions and mathematics competence generally followed the expected pattern. Enjoyment was positively associated with mathematics competence, whereas boredom and anxiety were negatively associated with competence. These associations were generally small, although most were statistically significant. The average variance extracted (AVE) values provided overall support for convergent validity. At the level of the specific dimensions, AVE values ranged from 0.47 to 0.77 at Wave 1 and from 0.52 to 0.79 at Wave 2. All but two values met or exceeded the recommended threshold of 0.50. The exceptions were anxiety in class (AVE = 0.48) and anxiety while learning (AVE = 0.47) at Wave 1, both of which fell only slightly below this criterion. At Wave 2, the AVE values for these dimensions increased to 0.54 and 0.52, respectively. For the higher-order dimensions, all AVE values were high, ranging from 0.82 to 0.91 at Wave 1 and from 0.82 to 0.90 at Wave 2. Overall, these findings support the reliability and convergent validity of the AEQ-ES scores at both measurement occasions.

## 4. Discussion

The present study examined the psychometric functioning of the AEQ-ES-Gn across two measurement occasions and across gender, grade, and country, as well as its temporal stability and associations with mathematics competence. The findings are discussed below in relation to the four research questions, distinguishing between cross-sectional and longitudinal evidence. Cross-sectional analyses conducted separately at each wave examined factorial structure, reliability, convergent and discriminant validity, and measurement invariance across gender, grade, and country. Longitudinal analyses examined measurement invariance across occasions and the stability of the corresponding achievement-emotion dimensions over approximately one year.

### 4.1. Structural Validity, Reliability, and Construct Distinctiveness (RQ1)

The present study aimed to assess the psychometric properties of a modified version of the Achievement Emotions Questionnaire–Elementary School (AEQ-ES; Lichtenfeld et al., 2012), referred to as the AEQ-ES-Gn. In this version, the original gender-specific pictorial response anchors were replaced with gender-neutral, smiley-based representations. The findings supported a hierarchical structure that differentiated enjoyment, boredom, and anxiety while also accounting for the academic settings in which these emotions were experienced. Models based solely on emotional valence or on the three broad emotion dimensions provided a less adequate representation of students’ responses, indicating that elementary school students’ responses differentiate not only among the three emotions but also among their class-, learning-, and test-related manifestations.

This factorial organisation is consistent with AEQ-ES validation research conducted across different linguistic, cultural, and educational contexts. The original German and US validation, the cross-national study involving Italy, Germany, and the United States, and the Japanese and Portuguese adaptations all supported a hierarchical structure accounting for both emotion and academic setting (Lichtenfeld et al., 2012; Raccanello et al., 2019; Sanches et al., 2020; Suzuki & Tonegawa, 2022). In studies that explicitly compared alternative factorial structures, the hierarchical model represented students’ responses more adequately than models based exclusively on emotional valence or broad emotion dimensions (Lichtenfeld et al., 2012; Raccanello et al., 2019; Suzuki & Tonegawa, 2022). This consistency accords with control-value theory, which conceptualises achievement emotions as both emotion-specific and context-specific (Pekrun, 2006, 2024; Pekrun & Perry, 2014), and with the taxonomy of achievement emotions, according to which enjoyment, boredom, and anxiety occupy distinct positions in terms of valence and activation (Pekrun et al., 2023). Although comparisons of model fit across studies should be interpreted cautiously because of differences in samples, estimation methods, treatment of clustering, and model specification, the present study extends previous evidence to a substantially larger and more culturally diverse group of students from six European countries.

The evaluation of a bifactor-like model represents an additional contribution of the present study. Although this model produced marginally better fit indices than the hierarchical model, it yielded inadmissible solutions during measurement invariance testing. Its slightly better global fit therefore did not translate into a sufficiently stable measurement structure across groups and occasions. The hierarchical model therefore offered a more parsimonious and interpretable representation that was also sufficiently stable for longitudinal and multigroup invariance testing and was consequently retained for subsequent analyses. This finding supports the representation adopted in previous validation studies, in which enjoyment, boredom, and anxiety are conceptualised as higher-order factors reflected by setting-specific emotional experiences (Lichtenfeld et al., 2012; Raccanello et al., 2019; Sanches et al., 2020; Suzuki & Tonegawa, 2022). More broadly, replication of this structure indicates that the established factorial organisation of the instrument was retained when the modified pictorial response formats were used. Because the two response formats were not compared directly, however, the findings support the adequacy of the modified anchors rather than their psychometric equivalence or superiority to the original format.

The reliability results were consistent with those reported in previous AEQ-ES validation studies across different linguistic and cultural contexts (Lichtenfeld et al., 2012; Raccanello et al., 2019; Sanches et al., 2020; Suzuki & Tonegawa, 2022). The original instrument demonstrated adequate to high internal consistency in German and US students (Lichtenfeld et al., 2012), and satisfactory reliability was subsequently reported in cross-national research and in the Portuguese and Japanese adaptations (Raccanello et al., 2019; Sanches et al., 2020; Suzuki & Tonegawa, 2022). Across these studies, the broader emotion dimensions tended to demonstrate stronger reliability than some of the narrower setting-specific dimensions (Lichtenfeld et al., 2012; Sanches et al., 2020). Similarly, in the present study, reliability was adequate to excellent at both waves and across composite reliability, Cronbach’s alpha, and McDonald’s omega, supporting the internal consistency of scores obtained using the modified pictorial response formats. Learning-related anxiety nevertheless remained less reliable than most other dimensions, a pattern also observed in previous research (Sanches et al., 2020). This may reflect greater situational variability in anxiety during learning activities, arising from differences in students’ control and value appraisals across tasks and learning situations (Pekrun, 2006, 2024), as well as the challenges involved in assessing a narrowly contextualised emotional experience with relatively few items.

The findings also provided overall support for the convergent validity of the AEQ-ES-Gn, as the specific and higher-order dimensions generally accounted for substantial variance in their respective indicators at both waves. Although previous validation studies did not always assess convergent validity using the same criteria, they reported well-defined factor structures and theoretically expected associations with control and value appraisals, perceived competence, motivation, learning strategies, and achievement (Lichtenfeld et al., 2012; Sanches et al., 2020; Suzuki & Tonegawa, 2022). The present findings therefore add evidence based on the amount of variance captured by the specific and higher-order dimensions.

Evidence concerning discriminant validity was more nuanced. The strong negative association between enjoyment and boredom is consistent with patterns reported across different linguistic and cultural contexts (Lichtenfeld et al., 2012; Raccanello et al., 2019; Sanches et al., 2020; Suzuki & Tonegawa, 2022), suggesting that the substantial overlap between the two emotions reflects their inherent relationship rather than an artefact of the modified pictorial response formats. Manifest and latent correlations between enjoyment and boredom were also examined separately by country at each wave. Although the magnitude of these associations differed significantly in some cross-country comparisons, enjoyment and boredom were consistently strongly negatively correlated in every country. Thus, the close relationship between the two emotions appears to be robust across countries, even though its strength varies across cultural contexts.

Control-value theory provides a possible explanation for this association, as both enjoyment and boredom are activity emotions that are particularly sensitive to students’ appraisals of an activity’s value. Enjoyment is more likely when an activity is perceived as valuable and students feel capable of engaging with it successfully, whereas boredom is more likely when the activity is perceived as lacking value or when its demands are poorly matched to students’ perceived competence (Pekrun, 2006, 2024; Pekrun & Perry, 2014). Consequently, the appraisal conditions that favour enjoyment tend to be incompatible with those that elicit boredom, which may account for their strong inverse association.

The strong negative association between enjoyment and boredom warrants careful interpretation. A high correlation between two latent dimensions indicates substantial shared variance but does not, by itself, establish that the dimensions are empirically indistinguishable. Enjoyment and boredom occupy theoretically distinct positions within the taxonomy of achievement emotions: enjoyment is a positive activating emotion, whereas boredom is a negative deactivating emotion (Pekrun et al., 2023). Consistent with this conceptual distinction, the AVE of each dimension exceeded their shared variance, and explicitly combining enjoyment and boredom into a single higher-order factor resulted in appreciably poorer model fit at both waves. Together, the theoretical distinction between the two emotions, the Fornell–Larcker criterion, and the comparative model results support retaining enjoyment and boredom as separate, albeit strongly related, constructs.

### 4.2. Measurement Invariance Across Gender, Grade and Country (RQ2)

The cross-sectional measurement invariance findings provide further evidence that the AEQ-ES-Gn functions consistently across gender, grade and country. Scalar invariance was supported across gender at both waves, indicating that the measurement parameters were comparable for boys and girls. This replicates previous evidence of gender invariance obtained using the original gender-specific anchors (Raccanello et al., 2019; Sanches et al., 2020) and is particularly relevant to the present adaptation, as one of its main purposes was to remove the need for students to identify with a male or female pictorial representation. Importantly, gender invariance demonstrates comparable measurement functioning across boys and girls, but should not itself be interpreted as evidence that the modified anchors are perceived as more inclusive by students; perceived inclusiveness was not directly assessed in the present study. Scalar invariance across grades also extends earlier evidence involving students at different stages of elementary education (Raccanello et al., 2019; Sanches et al., 2020). Given the developmental changes in reading ability, emotional understanding, metacognition, and familiarity with academic settings that occur during these years, the findings suggest that the AEQ-ES-Gn measures the same underlying constructs across the examined grade levels.

Cross-country scalar invariance was also supported, extending the original comparison between German and US students (Lichtenfeld et al., 2012) and the subsequent comparison involving Italy, Germany, and the United States (Raccanello et al., 2019) to six European countries. The negligible changes in fit following the imposition of equality constraints indicate that the factorial structure, factor loadings, and item intercepts were sufficiently comparable across the participating countries. Nevertheless, the absolute fit of the country models was weaker than that of the gender and grade models, suggesting that cross-country comparisons should be interpreted with some caution. Residual linguistic, cultural, or educational differences may affect particular items even when the overall invariance criteria are satisfied. Despite this qualification, the findings broaden the geographical and linguistic evidence supporting the use of the AEQ-ES-Gn in cross-cultural research.

### 4.3. Longitudinal Invariance and Temporal Stability (RQ3)

To our knowledge, the demonstration of longitudinal scalar invariance extends previous AEQ-ES validation research. Previous studies provided evidence of temporal stability through test–retest correlations (Sanches et al., 2020) but did not establish whether the measurement parameters remained equivalent across occasions. The present findings indicate that the factorial structure, factor loadings, and item intercepts were stable over a one-year interval. This distinction is important because test–retest correlations assess the relative stability of individual differences but do not demonstrate that the constructs are measured in the same way at different occasions. Longitudinal scalar invariance provides a stronger foundation for examining stability and change, allowing differences in latent means and longitudinal associations to be interpreted as reflecting substantive developmental or educational processes rather than changes in the functioning of the instrument.

The temporal stability findings extend the evidence provided by previous AEQ-ES validation research. Most earlier studies focused primarily on factorial structure, reliability, cross-group invariance, and associations with external variables (Lichtenfeld et al., 2012; Raccanello et al., 2019; Suzuki & Tonegawa, 2022), whereas longitudinal evidence concerning the stability of the AEQ-ES dimensions has been comparatively limited. The present results were broadly consistent with the moderate temporal stability previously observed over a shorter interval (Sanches et al., 2020), despite covering a full school year. This pattern suggests meaningful continuity in students’ achievement emotions while leaving considerable scope for change. Such a combination of stability and change is consistent with control-value theory, according to which achievement emotions are shaped by control and value appraisals that may show some continuity but can also change in response to students’ developing competence, achievement experiences, classroom environments, and academic demands (Pekrun, 2006, 2024; Pekrun & Perry, 2014).

Moreover, when all W1 dimensions were considered simultaneously, each dimension showed the strongest standardised association with its corresponding W2 outcome, providing evidence of construct-specific longitudinal continuity beyond the bivariate test–retest correlations. Together with longitudinal scalar invariance, these findings strengthen the evidence supporting the use of the instrument in longitudinal research.

### 4.4. Associations with Mathematics Competence (RQ4)

Associations with mathematics competence also converged with the criterion-related validity evidence reported across previous AEQ-ES validation studies. The original validation found that enjoyment was positively associated with mathematics achievement, whereas boredom and anxiety were negatively associated with it (Lichtenfeld et al., 2012). Subsequent research across countries and academic domains broadly reproduced this pattern, although the strength and statistical significance of the associations varied across emotions, settings, and domains (Raccanello et al., 2019). The Japanese adaptation further showed that enjoyment was positively related to control and value appraisals, intrinsic motivation, adaptive learning strategies, teacher support, and mathematics performance, whereas anxiety and boredom generally displayed the opposite pattern (Suzuki & Tonegawa, 2022). Related associations with perceived competence and autonomous motivation were also reported for the Portuguese adaptation (Sanches et al., 2020).

The present findings therefore replicate the broader nomological pattern predicted by control-value theory: enjoyment is generally associated with more favourable competence-related outcomes, whereas boredom and anxiety are associated with less favourable outcomes (Pekrun, 2006, 2024; Pekrun & Perry, 2014). However, the modest magnitude of the associations indicates that achievement emotions and mathematics competence are related but distinct constructs. This is theoretically plausible because academic performance is shaped by multiple cognitive, motivational, instructional, and contextual factors, while emotions and achievement may influence one another reciprocally over time (Forsblom et al., 2022; Lichtenfeld et al., 2023; Pekrun, 2024). Accordingly, these correlations provide evidence of criterion-related validity but should not be interpreted as indicating causal effects.

### 4.5. Limitations and Future Research

Although the study provides strong psychometric evidence for the AEQ-ES-Gn, several limitations warrant consideration. First, the gender-neutral, smiley-based pictorial anchors were not directly compared with the original gender-specific anchors. Consequently, the findings support the adequacy of the modified response format but do not establish its psychometric equivalence or superiority to the original format. Moreover, because the facial expressions convey emotion-specific information as well as response intensity, they may contribute to how students interpret the items rather than serving only as response anchors. The present study therefore evaluates the AEQ-ES-Gn with its pictorial format as a whole and does not determine the specific contribution of the facial expressions to students’ responses. Although the neutral facial sequences were developed through project-group revision and external advisory-group review, the perceived emotional content and intensity of the individual faces were not examined in a separate rating or cognitive validation study. Future studies could randomly assign students to the two response formats and examine differences in response distributions, measurement properties, comprehension, and perceived inclusiveness. They could also investigate how children interpret individual facial expressions and the intended progression of emotional intensity.

Second, the modified response format was validated only in the domain of mathematics. Although previous research has supported the use of the AEQ-ES in other academic domains (Raccanello et al., 2019), it remains necessary to establish whether the modified pictorial response formats function similarly when students report emotions related to other subjects, such as native-language learning or science. Such research would provide stronger evidence for the generalizability of the modified format across academic domains. Third, despite support for scalar invariance, the absolute fit of the country models was weaker than that of the gender and grade models. This suggests that linguistic, cultural, or educational differences may affect the functioning of particular items and warrants further investigation through differential item functioning analyses or country-specific qualitative studies. Fourth, the instrument does not assess test-related boredom, and some setting-specific factors are represented by relatively few items. This asymmetry limits comparisons of all three emotions across the same academic settings and may contribute to the comparatively lower reliability of certain dimensions. Future revisions could examine whether additional developmentally appropriate items improve the content coverage and measurement precision of these factors. Finally, the present validation covers students in Grades 3–5 and therefore does not provide evidence regarding the functioning of the modified pictorial response format in Grades 1–2. Although the original AEQ-ES has been used with younger elementary school students, the present findings should not be generalised to the full primary-school age range without further validation of the AEQ-ES-Gn among younger children. Such work should examine whether the item length, five-point response format, and modified pictorial anchors are developmentally appropriate for students in the earliest primary grades.

## 5. Conclusions

The findings of the present study indicate that the AEQ-ES retained satisfactory psychometric functioning when administered with gender-neutral, smiley-based pictorial anchors. The AEQ-ES-Gn replicated the established hierarchical structure and demonstrated satisfactory reliability, temporal stability, and theoretically consistent associations with mathematics competence. It also showed scalar invariance over time and across gender, grade, and six European countries. The study further extended previous validation research by comparing the hierarchical model with a bifactor-like alternative and by providing more detailed evidence of convergent and discriminant validity. These findings support the AEQ-ES-Gn as a reliable and valid instrument for assessing achievement emotions among students in grades 3–5 using a common non-gender-specific pictorial response format.

## Figures and Tables

**Table 1:** Goodness-of-Fit Indices for the Alternative AEQ-ES-GN Measurement Models at Waves 1 and 2.

		χ^2^	df	*p*	CFI	TLI	RMSEA with 95% CI	SRMR	AIC	BIC
Wave 1	Two-Factor Model	49,690.6	349	<0.001	0.685	0.658	0.110 [0.109, 0.111]	0.128	823,969.03	824,595.18
	Three-Factor Model	15,911.05	347	<0.001	0.901	0.892	0.062 [0.061, 0.063]	0.058	777,010.46	777,651.35
	Hierarchical Model	7284.62	339	<0.001	0.956	0.950	0.042 [0.041, 0.043]	0.052	762,926.39	763,626.20
	Bi-Factor Model	6777.38	322	<0.001	0.959	0.952	0.041 [0.041, 0.042]	0.050	761,853.79	762,678.84
Wave 2	Two-Factor Model	56,142.89	349	<0.001	0.666	0.639	0.123 [0.122, 0.124]	0.140	765,242.73	765,860.13
	Three-Factor Model	19,540.70	347	<0.001	0.885	0.875	0.072 [0.072, 0.073]	0.062	715,617.10	716,249.03
	Hierarchical Model	8381.07	339	<0.001	0.952	0.946	0.047 [0.047, 0.048]	0.056	699,196.92	699,886.96
	Bi-Factor Model	7507.98	322	<0.001	0.957	0.950	0.046 [0.045, 0.047]	0.055	697,706.88	698,520.40

*Note.* CFI = comparative fit index; TLI = Tucker–Lewis index; RMSEA = root mean square error of approximation; CI = confidence interval; SRMR = standardised root mean square residual; AIC = Akaike information criterion; BIC = Bayesian information criterion.

**Table 2:** Fit Indices for Longitudinal and Multigroup Measurement Invariance of the Hierarchical AEQ-ES-GN Model.

		χ^2^	df	*p*	CFI	TLI	RMSEA with 95% CI	ΔCFI	ΔRMSEA
Longitudinal	Configural	19,182.92	1425	<0.001	0.952	0.948	0.031 [0.031, 0.032]	-	-
	Metric	19,529.60	1450	<0.001	0.951	0.948	0.031 [0.031, 0.032]	0.001	0
	Scalar	20,103.87	1475	<0.001	0.950	0.947	0.031 [0.031, 0.032]	0.001	0
Gender									
Wave 1	Configural	7706.60	678	<0.001	0.955	0.950	0.042 [0.041, 0.043]	-	-
	Metric	7740.32	703	<0.001	0.955	0.952	0.041 [0.041, 0.042]	0	0.001
	Scalar	8514.80	731	<0.001	0.950	0.949	0.043 [0.042, 0.044]	0.005	−0.002
Wave 2	Configural	8870.47	678	<0.001	0.950	0.946	0.048 [0.047, 0.049]	-	-
	Metric	8911.20	703	<0.001	0.952	0.948	0.047 [0.046, 0.048]	−0.002	0.001
	Scalar	9909.11	731	<0.001	0.946	0.944	0.049 [0.048, 0.050]	0.006	−0.002
Country									
Wave 1	Configural	14,829.84	2239	<0.001	0.922	0.921	0.054 [0.053, 0.055]	-	-
	Metric	14,921.80	2264	<0.001	0.922	0.922	0.054 [0.053, 0.054]	0	0
	Scalar	15,397.15	2284	<0.001	0.919	0.92	0.054 [0.053, 0.055]	0.003	0
Wave 2	Configural	17,301.81	2240	<0.001	0.919	0.918	0.062 [0.061, 0.063]	-	-
	Metric	17,461.89	2265	<0.001	0.918	0.918	0.062 [0.061, 0.063]	0.001	0
	Scalar	18,039.20	2285	<0.001	0.915	0.916	0.062 [0.062, 0.063]	0.003	0
Grade									
Wave 1	Configural	7716.67	678	<0.001	0.954	0.949	0.042 [0.041, 0.043]	-	-
	Metric	7751.28	703	<0.001	0.954	0.951	0.041 [0.041, 0.042]	0	0.001
	Scalar	8224.05	731	<0.001	0.951	0.95	0.042 [0.041, 0.043]	0.003	−0.001
Wave 2	Configural	8829.07	681	<0.001	0.952	0.946	0.048 [0.047, 0.049]	-	-
	Metric	8952.25	706	<0.001	0.951	0.947	0.047 [0.046, 0.048]	0.001	0.001
	Scalar	9479.57	734	<0.001	0.948	0.946	0.048 [0.047, 0.048]	0.003	−0.001

*Note.* CFI = comparative fit index; TLI = Tucker–Lewis index; RMSEA = root mean square error of approximation; CI = confidence interval; ΔCFI = change in CFI; ΔRMSEA = change in RMSEA.

**Table 3:** Reliability and Convergent Validity Indices for the Specific and Higher-Order AEQ-ES-GN Dimensions at Waves 1 and 2.

			CR	Cronbach’s α	McDonald’s ω	AVE
Spc. Dim.	Wave 1	Enjoyment Class	0.93	0.93	0.93	0.77
		Enjoyment Learning	0.81	0.81	0.81	0.68
		Enjoyment Test	0.86	0.85	0.86	0.68
		Boredom Class	0.89	0.92	0.92	0.74
		Boredom Learning	0.89	0.89	0.90	0.74
		Anxiety Class	0.78	0.78	0.79	0.48
		Anxiety Learning	0.73	0.72	0.73	0.47
		Anxiety Test	0.90	0.90	0.90	0.66
	Wave 2	Enjoyment Class	0.94	0.94	0.94	0.79
		Enjoyment Learning	0.82	0.82	0.82	0.69
		Enjoyment Test	0.86	0.85	0.86	0.68
		Boredom Class	0.93	0.93	0.93	0.77
		Boredom Learning	0.90	0.90	0.90	0.76
		Anxiety Class	0.83	0.82	0.83	0.54
		Anxiety Learning	0.76	0.76	0.77	0.52
		Anxiety Test	0.92	0.92	0.92	0.69
H-O Dim.	Wave 1	Enjoyment	0.93	0.93	0.93	0.82
		Boredom	0.95	0.92	0.92	0.91
		Anxiety	0.95	0.78	0.79	0.87
	Wave 2	Enjoyment	0.93	0.94	0.94	0.82
		Boredom	0.95	0.93	0.93	0.90
		Anxiety	0.94	0.82	0.83	0.85

*Note.* CR = composite reliability; AVE = average variance extracted; α = Cronbach’s alpha; ω = McDonald’s omega; Spc. Dim. = specific dimensions; H-O Dim. = higher-order dimensions.

**Table 4:** Average variance extracted, correlations, and squared correlations among the higher-order dimensions of the AEQ-ES-GN.

		Enjoyment	Boredom	Anxiety
W1	Enjoyment	0.82	**−0.82**	−0.38
	Boredom	0.68	0.91	0.50
	Anxiety	0.15	0.25	0.87
W2	Enjoyment	0.82	**−0.84**	−0.38
	Boredom	0.70	0.90	0.49
	Anxiety	0.14	0.24	0.85

*Note.* W1 = Wave 1, W2 = Wave 2. Italicised values on the diagonal represent the average variance extracted. Values above the diagonal are correlations among the constructs, whereas values below the diagonal are squared correlations. All correlations are statistically significant at *p* < 0.001. Boldface indicates correlations with absolute values greater than 0.70.

**Table 5:** Test–retest correlations for the AEQ-ES-GN dimensions and their associations with Mathematics Competence.

	EnjCW2	EnjLW2	EnjTW2	BorCW2	BorLW2	AnxCW2	AnxLW2	AnxTW2	EnjW2	BorW2	AnxW2	T1 W1	T2 W1
EnjCW1	0.58 ***	0.49 ***	0.46 ***	−0.46 ***	−0.41 ***	−0.19 ***	−0.13 ***	−0.16 ***	0.56 ***	−0.46 ***	−0.18 ***	0.08 ***	0.16 ***
EnjLW1	0.52 ***	0.54 ***	0.42 ***	−0.41 ***	−0.41 ***	−0.12 ***	−0.09 ***	−0.11 ***	0.55 ***	−0.43 ***	−0.12 ***	−0.01	0.10 ***
EnjTW1	0.49 ***	0.43 ***	0.53 ***	−0.36 ***	−0.32 ***	−0.20 ***	−0.15 ***	−0.24 ***	0.54 ***	−0.36 ***	−0.22 ***	0.09 ***	0.18 ***
BorCW1	−0.46 ***	−0.38 ***	−0.34 ***	0.52 ***	0.46 ***	0.24 ***	0.18 ***	0.21 ***	−0.43 ***	0.51 ***	0.23 ***	−0.10 ***	−0.18 ***
BorLW1	−0.42 ***	−0.38 ***	−0.31 ***	0.48 ***	0.49 ***	0.21 ***	0.18 ***	0.18 ***	−0.41 ***	0.50 ***	0.21 ***	−0.07 ***	−0.14 ***
AnxCW1	−0.21 ***	−0.12 ***	−0.20 ***	0.26 ***	0.22 ***	0.49 ***	0.43 ***	0.41 ***	−0.19 ***	0.25 ***	0.49 ***	−0.23 ***	−0.21 ***
AnxLW1	−0.17 ***	−0.10 ***	−0.17 ***	0.22 ***	0.20 ***	0.42 ***	0.45 ***	0.40 ***	−0.16 ***	0.22 ***	0.47 ***	−0.20 ***	−0.19 ***
AnxTW1	−0.20 ***	−0.11 ***	−0.25 ***	0.26 ***	0.23 ***	0.44 ***	0.41 ***	0.52 ***	−0.21 ***	0.25 ***	0.52 ***	−0.21 ***	−0.22 ***
Enj_W1	0.58 ***	0.54 ***	0.52 ***	−0.46 ***	−0.42 ***	−0.19 ***	−0.13 ***	−0.19 ***	0.61 ***	−0.46 ***	−0.20 ***	0.06 ***	0.16 ***
Bor_W1	−0.46 ***	−0.40 ***	−0.34 ***	0.52 ***	0.50 ***	0.23 ***	0.19 ***	0.21 ***	−0.44 ***	0.53 ***	0.23 ***	−0.09 ***	−0.17 ***
Anx_W1	−0.22 ***	−0.13 ***	−0.24 ***	0.28 ***	0.25 ***	0.50 ***	0.48 ***	0.50 ***	−0.21 ***	0.27 ***	0.55 ***	−0.24 ***	−0.23 ***
T1 W2	0.10 ***	0.02	0.11 ***	−0.12 ***	−0.09 ***	−0.24 ***	−0.19 ***	−0.23 ***	0.09 ***	−0.11 ***	−0.24 ***	0.60 ***	0.48 ***
T2 W2	0.15 ***	0.09 ***	0.13 ***	−0.16 ***	−0.13 ***	−0.22 ***	−0.18 ***	−0.20 ***	0.13 ***	−0.15 ***	−0.23 ***	0.41 ***	0.86 ***

*Note.* W1 = Wave 1; W2 = Wave 2; Enj = enjoyment; Bor = boredom; Anx = anxiety; C = class-related; L = learning-related; T = test-related; T1 = Mathematics Competence Test 1; T2 = Mathematics Competence Test 2. Boldface indicates test–retest correlations between corresponding AEQ-ES-GN dimensions across waves. *** *p* < 0.001.

## Data Availability

Due to project-level restrictions, study data is not currently publicly available. De-identified datasets and codebooks will be stored in Zenodo and made accessible in phases starting autumn 2026 (access subject to the project’s governance procedures). All research instruments and code can be obtained from the research team.

## Supplementary Materials

The following supporting information can be downloaded at: https://www.mdpi.com/article/10.3390/bs16101928/s1, Title: Cross-Country Enjoyment–Boredom Correlations and Longitudinal Regression Analyses of AEQ-ES-Gn Dimensions. Table S1. Pearson Correlations and latent CFA correlations between enjoyment and boredom at Wave 1 and Wave 2 by Country. Table S2. Multiple Regression Predicting Enjoyment in Class at Wave 2 From AEQ-ES-Gn Dimensions at Wave 1. Table S3. Multiple Regression Predicting Enjoyment in Learning at Wave 2 from AEQ-ES-Gn Dimensions at Wave 1. Table S4. Multiple Regression Predicting Test Enjoyment at Wave 2 from AEQ-ES-Gn Dimensions at Wave 1. Table S5. Multiple Regression Predicting Boredom in Class at Wave 2 From AEQ-ES-Gn Dimensions at Wave 1. Table S6. Multiple Regression Predicting Boredom in Learning at Wave 2 from AEQ-ES-Gn Dimensions at Wave 1. Table S7. Multiple Regression Predicting Anxiety in Class at Wave 2 from AEQ-ES-Gn Dimensions at Wave 1. Table S8. Multiple Regression Predicting Anxiety in Learning at Wave 2 from AEQ-ES-Gn Dimensions at Wave 1. Table S9. Multiple Regression Predicting Test Anxiety at Wave 2 from AEQ-ES-Gn Dimensions at Wave 1.
