# Supplementary material for: Achievement Emotions Questionnaire–Elementary School: Psychometric Analysis of a Modified Version (AEQ-ES-Gn)
Source: Behav Sci (Basel). 2026 Oct 17;16(10):1928. doi: 10.3390/bs16101928 (PMC13701542; doi:10.3390/bs16101928)
Supplement: Supplementary file 1 [file behavsci-16-01928-s001.zip › behavsci-4553741-supplementary.pdf]

Correlations between Enjoyment and Boredom

Table S1. Pearson Correlations and latent CFA correlations between enjoyment and boredom at Wave 1 and Wave 2 by Country

Country	Pearson correlations W1	Latent correlations W1	Pearson correlations W2	Latent correlations W2
Estonia	-0.694[bcde]	-0.808[bcde]	-0.707[bcde]	-0.815[bcde]
Finland	-0.740[af]	-0.852[af]	-0.750[adf]	-0.850[adf]
Norway	-0.747[af]	-0.856 [af]	-0.758[adf]	-0.859[adf]
Portugal	-0.750[af]	-0.857 [af]	-0.796[abcef]	-0.913[abcef]
Serbia	-0.733[af]	-0.839 [af]	-0.753[adf]	-0.851[adf]
Sweden	-0.677 [abcde]	-0.804 [abcde]	-0.70[7abcde]	-0.828 [abcde]

Note. W1 = Wave 1; W2 = Wave 2. Superscript letters indicate statistically significant pairwise differences between countries within the same wave and correlation type ($p < .05$): a = Estonia, b = Finland, c = Norway, d = Portugal, e = Serbia, f = Sweden.

Regression Analyses Predicting AEQ-ES-Gn Dimensions at Wave 2

The following tables report eight multiple regression analyses. Across all models, the Wave 1 variable corresponding to the Wave 2 outcome had the largest standardized effect in absolute value.

Table S2. Multiple Regression Predicting Enjoyment in Class at Wave 2 From AEQ-ES-Gn Dimensions at Wave 1

Predictor	*B*	*SE B*	β	*t*	*p*
Constant	1.039	0.071	—	14.72	< .001
**Enjoyment in class (W1)**	**0.366**	**0.018**	**0.370**	**20.71**	**< .001**
Enjoyment in learning (W1)	0.131	0.014	0.136	9.18	< .001
Test enjoyment (W1)	0.111	0.013	0.113	8.53	< .001
Boredom in class (W1)	−0.012	0.021	-0.010	−0.59	= .557
Boredom in learning (W1)	−0.026	0.017	-0.024	−1.53	= .127
Anxiety in class (W1)	0.047	0.024	0.026	1.94	= .053
Anxiety in learning (W1)	−0.032	0.021	-0.020	−1.53	= .126
Test anxiety (W1)	−0.004	0.017	-0.003	−0.23	= .817

Note. N = 9,399. R^2 = .354, adjusted R^2 = .353; $F(8, 9{,}390) = 642.39$, $p < .001$. Boldface identifies the Wave 1 predictor corresponding to the Wave 2 outcome; in this model, it also has the largest standardized coefficient in absolute value. B = unstandardized regression coefficient; SE B = standard error of B; β = standardized regression coefficient; W1 = Wave 1; W2 = Wave 2.

Table S3. Multiple Regression Predicting Enjoyment in Learning at Wave 2 From AEQ-ES-Gn Dimensions at Wave 1

Predictor	*B*	*SE B*	β	*t*	*p*
Constant	0.493	0.071	—	6.99	< .001
Enjoyment in class (W1)	0.139	0.018	0.145	7.85	< .001
**Enjoyment in learning (W1)**	**0.328**	**0.014**	**0.350**	**22.91**	**< .001**
Test enjoyment (W1)	0.106	0.013	0.112	8.13	< .001
Boredom in class (W1)	0.038	0.021	0.033	1.79	= .073
Boredom in learning (W1)	−0.065	0.017	-0.063	−3.84	< .001
Anxiety in class (W1)	0.060	0.024	0.034	2.46	= .014
Anxiety in learning (W1)	−0.002	0.021	-0.001	−0.10	= .922
Test anxiety (W1)	0.055	0.017	0.045	3.30	< .001

Note. N = 9,375. R^2 = .312, adjusted R^2 = .311; $F(8, 9{,}366) = 530.23$, $p < .001$. Boldface identifies the Wave 1 predictor corresponding to the Wave 2 outcome; in this model, it also has the largest standardized coefficient in absolute value. B = unstandardized regression coefficient; SE B = standard error of B; β = standardized regression coefficient; W1 = Wave 1; W2 = Wave 2.

Table S4. Multiple Regression Predicting Test Enjoyment at Wave 2 From AEQ-ES-Gn Dimensions at Wave 1

Predictor	*B*	*SE B*	β	*t*	*p*
Constant	0.761	0.071	—	10.66	< .001
Enjoyment in class (W1)	0.145	0.018	0.151	8.10	< .001
Enjoyment in learning (W1)	0.069	0.014	0.074	4.79	< .001
**Test enjoyment (W1)**	**0.364**	**0.013**	**0.384**	**27.69**	**< .001**
Boredom in class (W1)	0.039	0.021	0.034	1.84	= .066
Boredom in learning (W1)	0.021	0.017	0.021	1.23	= .218
Anxiety in class (W1)	0.028	0.025	0.016	1.13	= .259
Anxiety in learning (W1)	−0.015	0.021	-0.009	−0.68	= .498
Test anxiety (W1)	−0.065	0.017	-0.053	−3.88	< .001

Note. *N* = 9,353. R^2 = .299, adjusted R^2 = .299; F(8, 9,344) = 498.93, p < .001. Boldface identifies the Wave 1 predictor corresponding to the Wave 2 outcome; in this model, it also has the largest standardized coefficient in absolute value. B = unstandardized regression coefficient; SE B = standard error of B; β = standardized regression coefficient; W1 = Wave 1; W2 = Wave 2.

Table S5. Multiple Regression Predicting Boredom in Class at Wave 2 From AEQ-ES-Gn Dimensions at Wave 1

Predictor	*B*	*SE B*	β	*t*	*p*
Constant	1.860	0.071	—	26.32	< .001
Enjoyment in class (W1)	−0.087	0.018	-0.092	−4.92	< .001
Enjoyment in learning (W1)	−0.065	0.014	-0.070	−4.55	< .001
Test enjoyment (W1)	−0.017	0.013	-0.018	−1.31	= .191
**Boredom in class (W1)**	**0.317**	**0.021**	**0.278**	**14.93**	**< .001**
Boredom in learning (W1)	0.112	0.017	0.110	6.59	< .001
Anxiety in class (W1)	−0.012	0.024	-0.007	−0.48	= .635
Anxiety in learning (W1)	−0.004	0.021	-0.002	−0.18	= .859
Test anxiety (W1)	0.080	0.017	0.066	4.82	< .001

Note. *N* = 9,400. R^2 = .289, adjusted R^2 = .288; F(8, 9,391) = 476.37, p < .001. Boldface identifies the Wave 1 predictor corresponding to the Wave 2 outcome; in this model, it also has the largest standardized coefficient in absolute value. B = unstandardized regression coefficient; SE B = standard error of B; β = standardized regression coefficient; W1 = Wave 1; W2 = Wave 2.

Table S6. Multiple Regression Predicting Boredom in Learning at Wave 2 From AEQ-ES-Gn Dimensions at Wave 1

Predictor	*B*	*SE B*	β	*t*	*p*
Constant	1.828	0.079	—	23.05	< .001
Enjoyment in class (W1)	−0.023	0.020	-0.023	−1.18	= .237
Enjoyment in learning (W1)	−0.128	0.016	-0.126	−7.95	< .001
Test enjoyment (W1)	0.003	0.015	0.003	0.24	= .811
Boredom in class (W1)	0.177	0.024	0.141	7.41	< .001
**Boredom in learning (W1)**	**0.294**	**0.019**	**0.264**	**15.42**	**< .001**
Anxiety in class (W1)	−0.032	0.027	-0.017	−1.19	= .235
Anxiety in learning (W1)	−0.003	0.024	-0.002	−0.13	= .897
Test anxiety (W1)	0.070	0.019	0.053	3.78	< .001

Note. N = 9,377. R^2 = .261, adjusted R^2 = .261; $F(8, 9{,}368) = 413.90$, $p < .001$. Boldface identifies the Wave 1 predictor corresponding to the Wave 2 outcome; in this model, it also has the largest standardized coefficient in absolute value. B = unstandardized regression coefficient; SE B = standard error of B; β = standardized regression coefficient; W1 = Wave 1; W2 = Wave 2.

Table S7. Multiple Regression Predicting Anxiety in Class at Wave 2 From AEQ-ES-Gn Dimensions at Wave 1

Predictor	*B*	*SE B*	β	*t*	*p*
Constant	0.693	0.048	—	14.49	< .001
Enjoyment in class (W1)	−0.019	0.012	-0.030	−1.55	= .121
Enjoyment in learning (W1)	0.011	0.010	0.017	1.09	= .276
Test enjoyment (W1)	0.001	0.009	0.001	0.10	= .918
Boredom in class (W1)	0.038	0.014	0.050	2.66	= .008
Boredom in learning (W1)	−0.045	0.011	-0.066	−3.89	< .001
**Anxiety in class (W1)**	**0.352**	**0.016**	**0.304**	**21.41**	**< .001**
Anxiety in learning (W1)	0.096	0.014	0.093	6.67	< .001
Test anxiety (W1)	0.137	0.011	0.170	12.20	< .001

Note. N = 9,400. R^2 = .264, adjusted R^2 = .263; $F(8, 9{,}391) = 420.07$, $p < .001$. Boldface identifies the Wave 1 predictor corresponding to the Wave 2 outcome; in this model, it also has the largest standardized coefficient in absolute value. B = unstandardized regression coefficient; SE B = standard error of B; β = standardized regression coefficient; W1 = Wave 1; W2 = Wave 2.

Table S8. Multiple Regression Predicting Anxiety in Learning at Wave 2 From AEQ-ES-Gn Dimensions at Wave 1

Predictor	*B*	*SE B*	β	*t*	*p*
Constant	0.626	0.052	—	11.94	< .001
Enjoyment in class (W1)	−0.007	0.013	-0.010	−0.52	= .606
Enjoyment in learning (W1)	0.014	0.011	0.021	1.32	= .187
Test enjoyment (W1)	0.011	0.010	0.016	1.13	= .257
Boredom in class (W1)	−0.002	0.016	-0.003	−0.14	= .890
Boredom in learning (W1)	−0.019	0.013	-0.026	−1.47	= .141
Anxiety in class (W1)	0.215	0.018	0.173	11.94	< .001
**Anxiety in learning (W1)**	**0.265**	**0.016**	**0.239**	**16.86**	**< .001**
Test anxiety (W1)	0.131	0.012	0.151	10.61	< .001

Note. N = 9,377. R^2 = .235, adjusted R^2 = .234; F(8, 9,368) = 359.86, p < .001. Boldface identifies the Wave 1 predictor corresponding to the Wave 2 outcome; in this model, it also has the largest standardized coefficient in absolute value. B = unstandardized regression coefficient; SE B = standard error of B; β = standardized regression coefficient; W1 = Wave 1; W2 = Wave 2.

Table S9. Multiple Regression Predicting Test Anxiety at Wave 2 From AEQ-ES-Gn Dimensions at Wave 1

Predictor	*B*	*SE B*	β	*t*	*p*
Constant	0.914	0.067	—	13.65	< .001
Enjoyment in class (W1)	0.018	0.017	0.021	1.09	= .276
Enjoyment in learning (W1)	0.026	0.014	0.030	1.94	= .052
Test enjoyment (W1)	−0.052	0.012	-0.059	−4.23	< .001
Boredom in class (W1)	0.066	0.020	0.061	3.26	= .001
Boredom in learning (W1)	−0.077	0.016	-0.080	−4.77	< .001
Anxiety in class (W1)	0.138	0.023	0.084	5.99	< .001
Anxiety in learning (W1)	0.075	0.020	0.052	3.75	< .001
**Test anxiety (W1)**	**0.482**	**0.016**	**0.424**	**30.67**	**< .001**

Note. N = 9,369. R^2 = .281, adjusted R^2 = .280; F(8, 9,360) = 457.14, p < .001. Boldface identifies the Wave 1 predictor corresponding to the Wave 2 outcome; in this model, it also has the largest standardized coefficient in absolute value. B = unstandardized regression coefficient; SE B = standard error of B; β = standardized regression coefficient; W1 = Wave 1; W2 = Wave 2.
